# Cognitive deficits profiles in the first-episode of schizophrenia, clinical high risk of psychosis, and genetically high-risk of psychosis

**DOI:** 10.3389/fpsyt.2023.1292141

**Published:** 2023-12-11

**Authors:** Fang Dong, Zhen Mao, Yushen Ding, Lu Wang, Qijing Bo, Feng Li, Feifei Wang, Chuanyue Wang

**Affiliations:** ^1^The National Clinical Research Center for Mental Disorders and Beijing Key Laboratory of Mental Disorders and Beijing Institute for Brain Disorders Center of Schizophrenia, Beijing Anding Hospital, Capital Medical University, Beijing, China; ^2^Advanced Innovation Center for Human Brain Protection, Capital Medical University, Beijing, China

**Keywords:** clinical high-risk for psychosis, genetically high-risk of psychosis, first episode schizophrenia, cognitive deficits, MCCB

## Abstract

**Background:**

Cognitive deficits are core characteristics of schizophrenia, presenting before the emergence of psychotic symptoms. Individuals with a clinical high-risk for psychosis (CHR) and those with genetically high-risk of psychosis (GHR) also exhibit cognitive impairments. Nonetheless, it remains uncertain in which domains of cognitive impairments in these two groups were more similar to those of schizophrenia patients. Moreover, it is unclear which domains of impairment are caused by quality factors and which are more related to the state of disease. This research initiative aimed to extensively examine the distinct cognitive impairment profiles among the CHR, GHR, and first-episode schizophrenia (FES) cohorts.

**Methods:**

We compared the cognitive functions of the three groups and a healthy control group (HCs) using the MATRICS Consensus Cognitive Battery (MCCB). The participants for this study were recruited from the Beijing Anding Hospital of Capital Medical University. Our sample consisted of 56 patients with FES, 42 with CHR, 26 with GHR, and 62 HCs. The participants across all groups were matched in terms of gender, age, and level of education.

**Results:**

Individuals with FES, GHR, and CHR showed significant impairment across the majority of MCCB domains, with the exception of visual learning, in comparison to HCs. None of the MCCB domains demonstrated a discerning ability to accurately differentiate between individuals with CHR and those with GHR. In the speed of processing and attention/vigilance domains, individuals with GHR and CHR exhibited scores between those of FES and HCs, with all group differences reaching statistical significance. This pattern of results indicates an intermediate level of cognitive function in individuals with GHR and CHR. Conversely, the levels of impairment observed in working memory and verbal learning were relatively consistent across all three groups: FES, CHR, and GHR. Notably, individuals in the CHR group exhibited performance akin to that of the HCs in the reasoning/problem-solving domain, while showing significant differences from the FES group, with the CHR individuals demonstrating better performance. Additionally, individuals with GHR displayed performance in social cognition similar to that of the HCs, while also demonstrating significant distinctions from the FES group, with the GHR individuals demonstrating better performance.

**Conclusion:**

Significant cognitive deficits exist in individuals with CHR, GHR, and FES, and these deficits vary across domains. Processing speed and attention/vigilance could potentially serve as robust biomarkers for identifying individuals at a risk of psychosis. The impairment observed in reasoning/problem-solving abilities might signify a qualitative trait, whereas deficits in social recognition could indicate a state characteristic specific to schizophrenia.

## 1 Introduction

Cognitive deficits are core characteristics of schizophrenia (1), and affect all aspects of neuropsychological functioning. Specifically, executive function, memory, and sustained attention seem to be particularly affected (2). Evidence suggests that cognitive decline precedes the emergence of psychotic symptoms (3), followed by a period of relative cognitive stability until later life (4).

Prior to the emergence of schizophrenia, many individuals experience non-specific symptoms such as perceptual disturbances, unusual beliefs or magical thinking, attentional disruptions, and symptoms of anxiety and depression. These manifestations are collectively denoted as clinical high-risk for psychosis (CHR) (5). Approximately one-third of individuals identified with CHR undergo a transition into psychosis within the subsequent 2–3 years (6). Compared to healthy controls (HCs), CHR individuals exhibit significant cognitive impairments, suggesting that neurocognitive dysfunction could serve as a potential biomarker for early detection and prognosis in this population (7).

Individuals who are first-degree relatives of schizophrenia patients, but currently show no clinical symptoms and function normally, are typically classified as genetically at high risk for psychosis (GHR). Individuals who are GHR demonstrate moderate cognitive deficits compared to healthy controls, and their cognitive profiles were similar to those observed in patients with schizophrenia (8, 9). Furthermore, those who are GHR for schizophrenia typically demonstrate poorer cognitive functioning than those at risk for affective psychosis. This observation implies that the genetic predisposition for schizophrenia, as marked by a positive GHR, exerts a significant influence on cognitive abilities (8).

Evidence indicates that considerable cognitive impairment among individuals with CHR is largely attributable to their transition to psychosis (CHR-T) (6). Therefore, neurocognitive deficits in CHR cohorts should be interpreted with caution, especially when considering psychosis or even CHR status as the specific clinical syndrome of interest, as these impairments likely signify a transdiagnostic or psychosis-specific vulnerability (10). It is important to note that the majority of CHR individuals do not develop psychosis (6). Consequently, the decrease in cognitive function might arise from either a subgroup genuinely at an elevated risk for psychosis who exhibits more pronounced impairments, or it might reflect generalized distress, psychopathology, or other psychiatric issues within CHR subjects (11). This highlights the importance of considering cognitive impairment among CHR subjects not solely as an exclusive marker for emerging psychosis, but potentially as a reflection of a broader range of underlying factors (12). Certain domains of cognitive impairment could potentially reflect qualitative traits associated with schizophrenia, rather than being indicative of current states. In these domains, the impairment in individuals with GHR may be more akin to that in the patient population than that observed in CHR individuals. Conversely, in domains where cognitive impairment represents a state characteristic, the impairment in CHR individuals could be more analogous to that in the patient population than in GHR individuals.

While existing literature generally acknowledges that both CHR and GHR individuals exhibit cognitive impairments compared with HCs, there is a paucity of studies that directly compare cognitive functioning across CHR, GHR, first-episode schizophrenia (FES) patients, and HCs (13, 14). Furthermore, previous research has not consistently utilized standardized cognitive assessment tools such as the MATRICS Consensus Cognitive Battery (MCCB) (13), or has only employed four of the seven cognitive domains assessed by the MCCB (14). MCCB was developed to provide a comprehensive assessment of cognitive functioning in patients with schizophrenia or schizoaffective disorder for the purposes of conducting clinical trials (15). Previous findings showed that the MCCB is a sensitive instrument to detect cognitive impairments in patients with schizophrenia (16–20).

In this study, we leveraged the MATRICS Consensus Cognitive Battery (MCCB) to compare the cognitive functions of individuals with FES, those at CHR, those with GHR, and HCs. Our objective was to explore the differences in cognitive profiles across these four groups. We aimed to pinpoint the shared domains of impairment across all three at-risk groups, and to highlight which domains of impairment are more pronounced within a particular group.

## 2 Materials and methods

This cross-sectional study was conducted between January 2015 and January 2018 at Beijing Anding Hospital of Capital Medical University. The study was reviewed and approved by the institutional ethics committee. All participants or their guardians in applicable cases provided their voluntary consent by signing written informed consent forms.

### 2.1 Participants

The study included individuals aged between 17 and 40 years, all of whom had completed at least an elementary education. FES patients were sourced from either outpatient services or inpatient wards, while those at the CHR were identified among the hospital’s help-seeking population. Individuals in the GHR and HCs were recruited through advertisements.

Patients with FES met the Diagnostic and Statistical Manual of Mental Disorders, Fourth Edition (DSM-IV) criteria for schizophrenia, with a first episode of disease and a duration of less than 3 years (21). These patients had either no history of medication or had used antipsychotics for no more than one continuous month since the onset of the disorder (22).

Individuals with CHR were screened using the Structured Interview for Psychosis-risk Syndromes (SIPS), qualifying if they met one or more of three conditions: Brief Intermittent Psychotic Symptoms Syndrome (BIPS), Attenuated Psychotic Symptoms Syndrome (APSS), or Genetic Risk and Deterioration Syndrome (GRD) (23).

Individuals with GHR were defined as first-degree relatives (siblings or children) of individuals diagnosed with schizophrenia. Any psychiatric disorders in individuals with GHR and HCs were ruled out using the Structured Clinical Interview for DSM-IV Axis I Disorders-Patient Edition (SCID-I/P) and SIPS. If a GHR individual meets the criterion of more than 30% functional deterioration in the past year as defined by the SIPS, they are included in the CHR group.

Participants were excluded if they had a severe physical illness or had undergone modified electroconvulsive therapy within the past 6 months. Substance-induced schizophrenia and patients with organic brain disorders were excluded from the study.

### 2.2 Measures

#### 2.2.1 Clinical assessment

The severity of symptoms in patients with FES was evaluated using the Positive and Negative Syndrome Scale (PANSS). This scale consists of 30 items, each with a defined criterion and a specific seven-level operational scoring standard (ranging from 1 to 7) (24).

To assess symptom scores for CHR, GHR, and HC individuals, we used the Scale of Prodromal Symptoms (SOPS) included in the Structured Interview for Psychosis-risk Syndromes (SIPS). The SOPS comprises 19 fundamental items, each rated on a seven-point scale (ranging from 0 to 6) (23).

#### 2.2.2 Cognitive function assessment

The MATRICS Consensus Cognitive Battery (MCCB) was utilized to assess the neurocognitive levels of the participants (25). It encompasses 10 subtests that measure seven cognitive domains: information processing speed, attention/vigilance, working memory, verbal learning, visual learning, reasoning and problem-solving, and social cognition. This study employed the Chinese version of the MCCB (26). The assessors conducting the evaluations underwent training from the staff at the Institute of Mental Health of Peking University, who participated in the development of the Chinese version of the MCCB. Subsequently, the raw scores were converted into T-scores using gender and age corrections based on the Chinese cognitive norms, with higher T-scores indicating superior cognitive function.

#### 2.2.3 Statistical analysis

The data were analyzed using IBM SPSS Statistics 23.0 for Windows (SPSS, Inc., Chicago, IL, United States). Continuous variables are presented as means and standard deviations, while categorical variables are presented as frequencies and percentages. Differences in demographic data between groups were assessed using the chi-square test or one-way ANOVA. Differences in cognitive domains among the four groups were analyzed using a Multivariate Analysis of Covariance (MANCOVA), with gender, age, years of education, and unemployment status as covariates. Analysis of Covariance (ANCOVA) was used to compare the overall composite scores among the four groups. *Post hoc* comparisons were conducted using Bonferroni correction. Effect sizes (Cohen’s *d*) were calculated to identify differences in cognitive performance levels. A value of *p* of less than 0.05 was deemed to represent statistical significance.

## 3 Results

### 3.1 Demographics and clinical characteristics

During the initial screening, five individuals with FES and two individuals with CHR were excluded because of non-cooperation with cognitive testing. Ultimately, a cohort of 186 Chinese participants was enrolled, consisting of 56 FES patients, 42 CHR individuals, 26 GHR individuals, and 62 HCs (Refer to Table 1). No significant differences were observed across the four groups in terms of age, years of education, sex ratio, marital status, or smoking status. However, the FES group had a significantly higher unemployment rate than the other three groups (χ^2^ = 28.51, *p* < 0.001). Additionally, all SOPS scores in the CHR group were significantly higher than those in the GHR and HC groups (χ^2^ = 94.06, *p* < 0.001).

**Table 1:** Demographics and clinical features of the participants.^*^

	FES	CHR^*^	GHR	HCs	Total	*F*	*p*
Subjects, *n*	56	42	26	62	186	—	—
Age, years	25.7 ± 6.5	23.8 ± 4.8	26.7 ± 4.8	25.1 ± 3.6	25.2 ± 5.1	2.07	0.11
Education, years	12.9 ± 3.2	14.3 ± 2.9	13.2 ± 3.2	14.2 ± 3.3	13.7 ± 3.2	2.60	0.05
Duration of illness, months	27.4 ± 26.2	26.3 ± 27.8	—	—	27.0 ± 26.7	0.04	0.84
SIPS							
Positive	—	9.4 ± 4.1	0.3 ± 0.5	0.3 ± 1.2	—	96.55	< 0.001
Negative	—	9.0 ± 5.2	0.7 ± 1.6	0.2 ± 0.8	—	95.49	< 0.001
Disorganization	—	4.7 ± 3.4	0.5 ± 0.8	0.1 ± 0.5	—	84.72	< 0.001
General	—	4.9 ± 3.5	0.8 ± 1.5	0.1 ± 0.5	—	83.59	< 0.001
Total score	—	28.0 ± 12.4	2.2 ± 3.7	0.8 ± 2.6	—	94.06	< 0.001
PANSS							
Positive	22.8 ± 6.1	—	—	—	—	—	—
Negative	21.0 ± 8.3	—	—	—	—	—	—
General psychopathology	41.9 ± 6.7	—	—	—	—	—	—
Total score	84.3 ± 15.0	—	—	—	—	—	—
						χ^2^	*p*
Men	30 (53.6)	26 (61.9)	15 (57.7)	35 (56.5)	106 (57.0)	0.69	0.88
Married	11 (19.6)	5 (11.9)	8 (30.8)	10 (16.1)	34 (18.3)	4.12	0.25
Family history^*^	9 (16.1)	12 (28.6)^*^	26(100.0)	0(0%)	47 (25.3)	100.61	< 0.001
Smoking	8 (14.3)	6 (14.3)	3 (11.5)	9 (14.5)	26 (14.0)	0.16	0.98
Unemployed	24 (42.9)	6 (14.3)	4 (15.4)	3 (4.8)	37 (19.9)	28.51	< 0.001
Medication	48 (85.7)	24 (57.1)	—	—	72	—	—
Unmedicated	8 (14.3)	18 (42.9)	—	—	26	—	—
Only AP^*^	44 (78.6)	13 (31.0)	—	—	57	—	—
Only AD^*^	0	5 (11.9)	—	—	5	—	—
AD + AP	1 (1.8)	4 (9.5)	—	—	5	—	—
Unspecified	3 (5.3)	2 (4.7)	—	—	5	—	—

^*^Data are reported as *n* (%), unless indicated otherwise. 37 cases of APS, two cases of BLIPS, and three cases of GRD were included in the CHR group. The family history refers to the presence of mental illness in the relatives of the subjects in two families and three generations. The 12 people listed in the family history of CHR are “first-degree relatives who had a family history of psychosis.” AD, Antidepressant; AP, Antipsychotic.

### 3.2 Comparison of cognitive performance among study groups

#### 3.2.1 FES, CHR, and GHR groups vs. healthy controls

No significant differences were observed in the MANCOVA of the visual learning domain (*F* = 1.96, *p* = 0.12). However, significant differences between the groups were noted in the remaining cognitive domains and overall composite scores (Table 2).

**Table 2:** Cognitive functions of the FES, CHR, GHR, and HCs.

Domains	FES	CHR	GHR	HCs	Total	Statistic^a^		Pairwise comparison ^b^		
						*F*	*p*		*p*	*Effect size ^c^*
Speed of processing	33.0 ± 8.9	39.0 ± 7.6	40.6 ± 5.1	45.2 ± 6.9	39.5 ± 8.9	15.72	< 0.001	FES < CHR	0.008	0.73
								FES < GHR	0.04	1.09
								FES < HC	< 0.001	1.54
								CHR < HC	< 0.001	0.86
								GHR < HC	0.006	0.77
Attention/Vigilance	30.1 ± 10.1	40.8 ± 11.3	39.4 ± 8.1	46.0 ± 8.5	39.3 ± 11.5	18.69	< 0.001	FES < CHR	< 0.001	1.00
								FES < GHR	0.001	1.02
								FES < HC	< 0.001	1.71
								CHR < HC	0.03	0.53
								GHR < HC	0.004	0.80
Working memory	38.5 ± 9.7	39.1 ± 3.4	42.3 ± 17.0	46.6 ± 6.9	41.9 ± 11.6	5.88	0.001	FES < HC	0.001	0.98
								CHR < HC	0.001	1.46
								GHR < HC	0.016	0.36
Verbal learning	38.7 ± 9.0	42.2 ± 9.6	40.8 ± 6.2	46.9 ± 10.6	42.5 ± 9.9	5.15	0.002	FES < HC	0.001	0.84
								CHR < HC	0.007	0.47
								GHR < HC	0.005	0.73
Visual learning	39.3 ± 14.1	42.8 ± 11.8	44.9 ± 9.9	47.1 ± 10.3	43.5 ± 12.2	1.96	0.12	—	—	—
Reasoning/problem solving	34.4 ± 11.0	40.7 ± 11.3	37.6 ± 8.4	43.4 ± 10.5	39.3 ± 11.2	3.87	0.01	FES < CHR	0.018	0.57
								FES < HC	0.002	0.84
Social recognition	31.4 ± 12.3	36.6 ± 8.1	39.7 ± 10.4	39.3 ± 9.8	36.4 ± 10.8	3.92	0.01	FES < GHR	0.004	0.73
								FES < HC	0.003	0.71
Overall composite	35.4 ± 6.4	40.6 ± 5.8	41.2 ± 3.0	45.0 ± 5.7	40.7 ± 6.9	7.00	< 0.001	FES < HC	< 0.001	1.59
								CHR < HC	0.004	0.77
								GHR < HC	0.031	0.87

^a^Multivariate analysis of covariance. ^b^Bonferroni correction applied to *post hoc* pairwise comparisons analyses. ^c^After significant pairwise comparisons, effect sizes were calculated using *Cohen’s d*.

**Figure 1:**
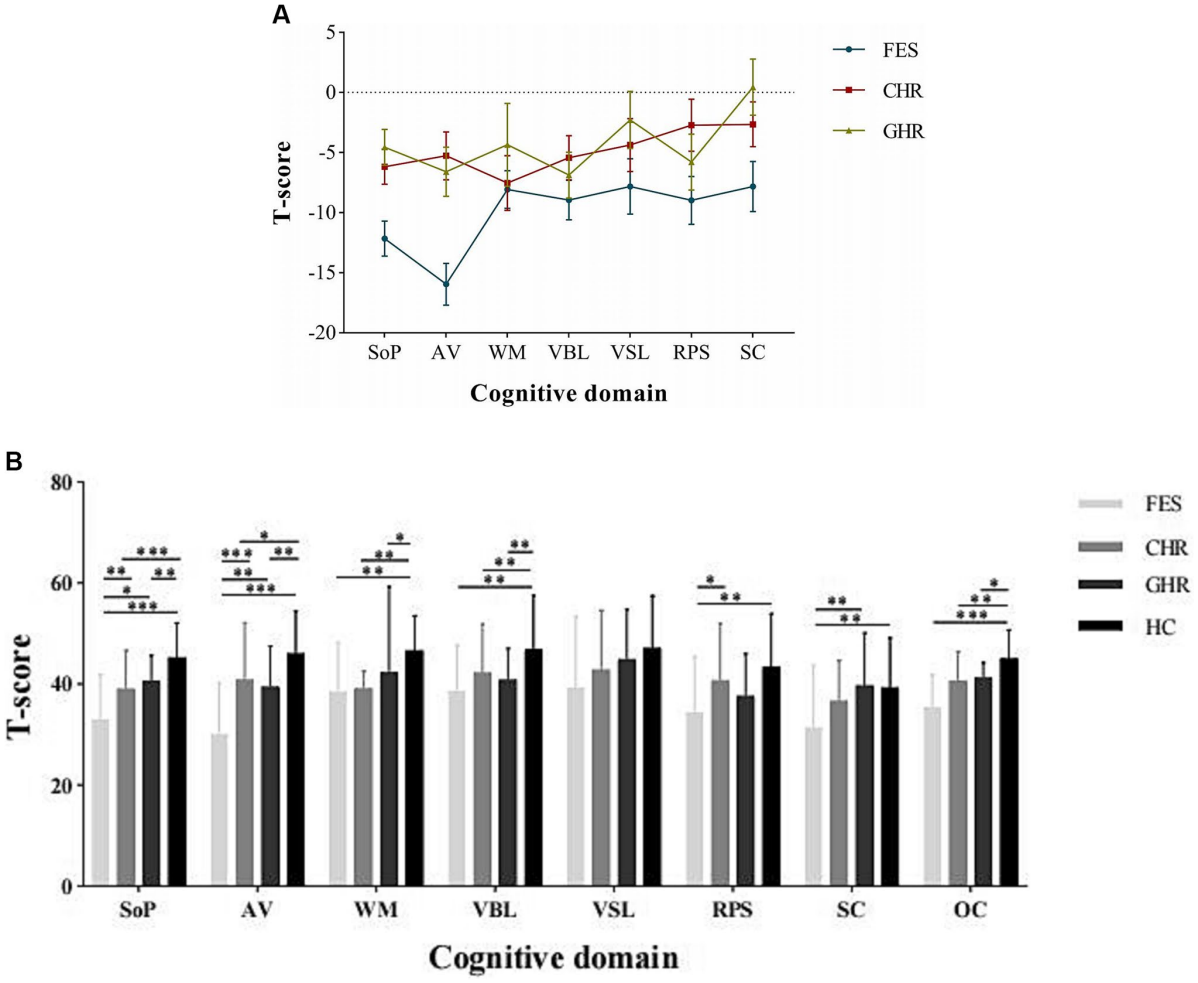
Cognitive profiles of the FES, CHR, and GHR groups against HC group. FES, First-episode schizophrenia; CHR, Clinical high-risk for psychosis; GHR, Genetically high-risk of psychosis; HC, healthy control. SoP, Speed of processing; AV, Attention/Vigilance; WM, Working memory; VBL, Verbal learning; VSL, Visual learning; RPS, Reasoning and problem solving; SC, Social cognition; and OC, Overall composite. The Y-axis in presents the mean and standard error of the difference between the study group and HC group.

*Post hoc* comparisons demonstrated that the performance of individuals with FES was significantly inferior to that of HCs in six of the seven cognitive domains, with the exception of visual learning (*Cohen’ d* = 0.71–1.71). Compared to the HCs, both the CHR (*Cohen’ d* = 0.47–1.46) and GHR (*Cohen’ d* = 0.36–1.80) groups exhibited significantly worse performance in the domains of information processing speed, attention/vigilance, working memory, verbal learning, and the overall composite score. The cognitive profiles of the FES, CHR, and GHR groups compared to those of the HC group are shown in Figure 1.

#### 3.2.2 Comparison between FES group and CHR group

First-episode schizophrenia patients scored lower than CHR individuals in the domains of information-processing speed (*p* = 0.008, *Cohen’d* = 0.73), attention/vigilance (*p* < 0.001, *Cohen’d* = 1.00), and reasoning/problem solving (*p* = 0.018, *Cohen’d* = 0.57).

#### 3.2.3 Comparison between FES group and GHR group

First-episode schizophrenia patients performed worse than GHR individuals in the domains of information processing speed (*p* = 0.04, *Cohen’d* = 1.09), attention/vigilance (*p* = 0.001, *Cohen’d* = 1.02), and social cognition (*p* = 0.004, *Cohen’d* = 0.73).

#### 3.2.4 Comparison between CHR group and GHR group

No significant differences were observed in the performance across all cognitive domains between the CHR and GHR groups.

## 4 Discussion

In this study, cognitive performance in the FES, GHR, CHR, and HC groups was investigated. Our findings indicate that individuals in the FES, GHR, and CHR groups exhibited notably poorer performance across the majority of domains assessed by the MCCB, in contrast to the HC group. While cognitive impairment was evident in both GHR and CHR individuals, its severity was milder than that observed in patients with FES.

In our study, cognitive functioning in individuals with CHR occupied an intermediate position between that of HCs and FES, which is consistent with previous research (14, 27). CHR individuals exhibited lower performance than HCs across all MCCB domains except for visual learning, especially in the domains of processing speed and attention/vigilance. Previous meta-analyses have consistently noted that CHR subjects lag behind HCs in all MCCB cognitive domains, particularly processing speed, attention/vigilance, and working memory (28). The cognitive deficit domains identified in the CHR group in our study echo those found in previous studies.

Several studies have compared cognitive impairment among the FES, CHR, and GHR groups (13, 14, 29). In a previous study (12), no significant differences were observed in the cognitive performances between the ultra-high risk (UHR) for psychosis and familial high-risk group (FHR) groups. The cognitive deficits in the UHR and FHR groups were largely similar to those in the FES group. Nonetheless, another study reached a different conclusion, suggesting that the performance in psychomotor speed, attention, processing speed and working memory, and verbal memory gradually decreased from the HC, first-degree relatives (FDR), UHR to FES groups (14). This implies that cognitive functioning in the UHR group was intermediate between that in the FES and FDR groups. The findings of our study appear to be more aligned with the first study mentioned earlier (13). Our study and the first study mentioned share similarities in the distribution of sample sizes across groups (our sample sizes: FES = 56, CHR = 42, and GHR = 26; the first study’s sample sizes: FES = 53, CHR = 52, and GHR = 29). However, the latter study mentioned had a larger sample size in the GHR group (FES = 40, CHR = 40, and GHR = 40) compared to ours (14). We wonder if our smaller GHR group size might have masked a potentially modest difference between the GHR and CHR groups, which warrants further investigation to confirm.

In our study, we detected impaired processing speed, attention/vigilance, working memory, and verbal learning in the GHR group, with the most significant impairments observed in the attention/vigilance domain. This finding aligns with previous studies that have suggested that individuals with GHR exhibit cognitive impairments resembling their affected siblings and demonstrate moderate deficits compared with HCs (8, 30–32). A quantitative and qualitative review has reported larger effect sizes for measures of full-scale IQ, vocabulary, and single-word reading tests, while measures of declarative memory, sustained attention, and working memory showed more modest effect sizes (8). The differences observed in the cognitive impairment domains between our study and previous studies may be attributed to variations in the assessment tools used. Different assessment tools measure various domains, or the same assessment item may be considered to reflect different domains in different assessment toolkits. For instance, in the aforementioned review, the IQ measures are typically composed of more elemental measures such as processing speed, working memory, language ability, and visual–spatial ability. The mentioned severe impairment in full-scale IQ in these studies also implies serious impairments in processing speed and working memory. These apparent differences might essentially be the same at their core. Similarly, the systemic review also mentions severe impairment in vocabulary, which actually corresponds to verbal learning in our study. This is also a key reason why recent studies have increasingly standardized the use of the MATRICS Consensus Cognitive Battery (MCCB), as it allows for comparability between research findings.

Among the three groups (FES, CHR, and GHR), processing speed and attention/vigilance were consistently impaired, with CHR and GHR individuals exhibiting milder impairments than FES individuals. This finding is in line with those of previous studies (29, 33). These results suggest that processing speed and attention/vigilance could potentially serve as promising biomarkers for early detection and severity assessment of schizophrenia. We hypothesized that genetic factors, current symptoms, or other unknown factors may influence these cognitive domains, with their effects potentially accumulating over time. Consequently, the most pronounced impairment in these particular domains was noted within the FES group.

Interestingly, the severity of impairment in the reasoning/problem-solving domain was comparable between GHR and FES (with no statistically significant difference), while CHR exhibited milder impairment than FES (with a statistically significant difference). On the other hand, the severity of impairment in social recognition was similar between CHR and FES (with no statistically significant difference), while GHR displayed less impairment than FES (with a statistically significant difference). Previous studies have consistently reported impaired social cognition in in individuals with CHR (34). Research on social cognition in individuals with GHR has been limited and inconsistent. However, previous findings have indicated that social cognitive impairments are significantly associated with psychopathology in young relatives of individuals with schizophrenia (35). Building on these insights, we propose that social recognition could potentially be more closely tied to an individual’s current state, while reasoning/problem-solving may be more indicative of qualitative differences.

The utilization of the MCCB in this study contributed to standardized cognitive testing and domains. Nevertheless, it is crucial to interpret these results with caution because of several limitations. First, the sample size was relatively small, which may have limited the generalizability of the findings. Second, the cross-sectional design of the study prevented the determination of predictive neuropsychological markers for the transition to psychosis in at-risk individuals. Third, the family history of the 12 individuals with CHR may serve as a confounding factor. Subsequent analyses could benefit from an expanded sample size and the incorporation of longitudinal observations from clinical and genetic high-risk psychosis cohorts to fortify the robustness of the findings. Additionally, antipsychotic medications may potentially account for the cognitive impairments. We were unable to exclusively collect data from unmedicated patients and have strived to minimize the impact of medications by including patients who have not been on regular medication for over 1 month.

## 5 Conclusion

Our study provides evidence supporting the existence of cognitive deficits in individuals at high risk for schizophrenia, both in clinical (CHR) and genetic (GHR) predispositions, prior to the onset of the first episode. Notably, processing speed and attention/vigilance emerged as shared domains that exhibited progressive impairment across the three groups, indicating their potential as biomarkers for schizophrenia. The observed impairment in reasoning/problem solving might signify a qualitative trait, whereas social recognition could potentially reflect an individual’s current state. However, it is crucial to emphasize that additional rigorous research is necessary to validate and substantiate these findings.

## Data availability statement

The original contributions presented in the study are included in the article/supplementary material, further inquiries can be directed to the corresponding author.

## Ethics statement

The studies involving humans were approved by Beijing Anding Hospital Ethics Committee. The studies were conducted in accordance with the local legislation and institutional requirements. The participants provided their written informed consent to participate in this study.

## Author contributions

FD: Investigation, Methodology, Project administration, Writing – original draft, Writing – review & editing. ZM: Data curation, Formal analysis, Investigation, Software, Writing – review & editing. YD: Formal analysis, Writing – review & editing. LW: Writing – review & editing. QB: Methodology, Project administration, Supervision, Writing – review & editing. FL: Investigation, Writing – review & editing. FW: Investigation, Writing – review & editing. CW: Project administration, Resources, Supervision, Writing – review & editing.

## References

[1] Bora E Yucel M Pantelis C. Cognitive impairment in schizophrenia and affective psychoses: implications for DSM-V criteria and beyond. Schizophr Bull. (2010) 36:36–42. doi: 10.1093/schbul/sbp094, PMID: 19776206 PMC2800141

[2] Jauhar S Johnstone M McKenna PJ. Schizophrenia. Lancet. (2022) 399:473–86. doi: 10.1016/S0140-6736(21)01730-X 35093231

[3] Lewandowski KE Cohen BM Ongur D. Evolution of neuropsychological dysfunction during the course of schizophrenia and bipolar disorder. Psychol Med. (2011) 41:225–41. doi: 10.1017/S0033291710001042 20836900

[4] Palmer BW Dawes SE Heaton RK. What do we know about neuropsychological aspects of schizophrenia? Neuropsychol Rev. (2009) 19:365–84. doi: 10.1007/s11065-009-9109-y, PMID: 19639412 PMC2745531

[5] Bo Q Mao Z Zhao L Li W Sun Y Wang C. Evolution of terms and concepts associated with clinical high risk psychosis. Chin J Psychiatry. (2019) 52:420–1. doi: 10.3760/cma.j.issn.1006-7884.2019.06.012

[6] Fusar-Poli P Bonoldi I Yung AR Borgwardt S Kempton MJ Valmaggia L. Predicting psychosis: meta-analysis of transition outcomes in individuals at high clinical risk. Arch Gen Psychiatry. (2012) 69:220–9. doi: 10.1001/archgenpsychiatry.2011.1472, PMID: 22393215

[7] Catalan A Salazar DPG Aymerich C Damiani S Sordi V Radua J. Neurocognitive functioning in individuals at clinical high risk for psychosis: a systematic review and Meta-analysis. JAMA Psychiatry. (2021) 78:859–67. doi: 10.1001/jamapsychiatry.2021.1290, PMID: 34132736 PMC8209603

[8] Agnew-Blais J Seidman LJ. Neurocognition in youth and young adults under age 30 at familial risk for schizophrenia: a quantitative and qualitative review. Cogn Neuropsychiatry. (2013) 18:44–82. doi: 10.1080/13546805.2012.676309, PMID: 22998599 PMC3577989

[9] Vyas NS Burke L Netherwood S Caviston P Simic M Buchsbaum MS. Neurocognitive profile of adolescents with early-onset schizophrenia and their unaffected siblings. World J Biol Psychiatry. (2022) 23:677–88. doi: 10.1080/15622975.2021.2023758, PMID: 34989324

[10] Millman ZB Roemer C Vargas T Schiffman J Mittal VA Gold JM. Neuropsychological performance among individuals at clinical high-risk for psychosis vs putatively low-risk peers with other psychopathology: a systematic review and Meta-analysis. Schizophr Bull. (2022) 48:999–1010. doi: 10.1093/schbul/sbac031, PMID: 35333372 PMC9434467

[11] Velthorst E Nieman DH Becker HE van de Fliert R Dingemans PM Klaassen R. Baseline differences in clinical symptomatology between ultra high risk subjects with and without a transition to psychosis. Schizophr Res. (2009) 109:60–5. doi: 10.1016/j.schres.2009.02.002, PMID: 19272756

[12] Lin A Yung AR Nelson B Brewer WJ Riley R Simmons M. Neurocognitive predictors of transition to psychosis: medium- to long-term findings from a sample at ultra-high risk for psychosis. Psychol Med. (2013) 43:2349–60. doi: 10.1017/S0033291713000123, PMID: 23388122

[13] Ucok A Direk N Koyuncu A Keskin-Ergen Y Yuksel C Guler J. Cognitive deficits in clinical and familial high risk groups for psychosis are common as in first episode schizophrenia. Schizophr Res. (2013) 151:265–9. doi: 10.1016/j.schres.2013.10.030, PMID: 24262680

[14] Hou CL Xiang YT Wang ZL Everall I Tang Y Yang C. Cognitive functioning in individuals at ultra-high risk for psychosis, first-degree relatives of patients with psychosis and patients with first-episode schizophrenia. Schizophr Res. (2016) 174:71–6. doi: 10.1016/j.schres.2016.04.034, PMID: 27197904

[15] Nuechterlein KH Green MF Kern RS Baade LE Barch DM Cohen JD. The Matrics consensus cognitive battery, part 1: test selection, reliability, and validity. Am J Psychiatry. (2008) 165:203–13. doi: 10.1176/appi.ajp.2007.07010042, PMID: 18172019

[16] Keefe RS Fox KH Harvey PD Cucchiaro J Siu C Loebel A. Characteristics of the Matrics consensus cognitive battery in a 29-site antipsychotic schizophrenia clinical trial. Schizophr Res. (2011) 125:161–8. doi: 10.1016/j.schres.2010.09.015, PMID: 21075600

[17] Kern RS Gold JM Dickinson D Green MF Nuechterlein KH Baade LE. The Mccb impairment profile for schizophrenia outpatients: results from the Matrics psychometric and standardization study. Schizophr Res. (2011) 126:124–31. doi: 10.1016/j.schres.2010.11.008, PMID: 21159492 PMC3050090

[18] Shamsi S Lau A Lencz T Burdick KE DeRosse P Brenner R. Cognitive and symptomatic predictors of functional disability in schizophrenia. Schizophr Res. (2011) 126:257–64. doi: 10.1016/j.schres.2010.08.007, PMID: 20828991 PMC3050077

[19] Lystad JU Falkum E Haaland V Bull H Evensen S Bell MD. Ueland T. Neurocognition and occupational functioning in schizophrenia Spectrum disorders: the Matrics consensus cognitive battery (Mccb) and workplace assessments. Schizophr Res. (2016) 170:143–9. doi: 10.1016/j.schres.2015.12.002, PMID: 26692347

[20] McCleery A Ventura J Kern RS Subotnik KL Gretchen-Doorly D Green MF. Cognitive functioning in First-episode schizophrenia: Matrics consensus cognitive battery (Mccb) profile of impairment. Schizophr Res. (2014) 157, 157:33–9. doi: 10.1016/j.schres.2014.04.039, PMID: 24888526 PMC4112962

[21] First MB SRGM (2012). Structured clinical interview for DSM-IV Axis I disorders: Corsini encyclopedia of psychology.

[22] Cheng Z Yuan Y Han X Yang L Cai S Yang F. An open-label randomised comparison of aripiprazole, olanzapine and risperidone for the acute treatment of First-episode schizophrenia: eight-week outcomes. J Psychopharmacol. (2019) 33:1227–36. doi: 10.1177/0269881119872193, PMID: 31487208

[23] Miller TJ McGlashan TH Rosen JL Cadenhead K Cannon T Ventura J. Prodromal assessment with the structured interview for prodromal syndromes and the scale of prodromal symptoms: predictive validity, interrater reliability, and training to reliability. Schizophr Bull. (2003) 29:703–15. doi: 10.1093/oxfordjournals.schbul.a007040, PMID: 14989408

[24] Kay SR Fiszbein A Opler LA. The positive and negative syndrome scale (PANSS) for schizophrenia. Schizophr Bull. (1987) 13:261–76. doi: 10.1093/schbul/13.2.261 3616518

[25] Kern RS Nuechterlein KH Green MF Baade LE Fenton WS Gold JM. The MATRICS consensus cognitive battery, part 2: co-norming and standardization. Am J Psychiatry. (2008) 165:214–20. doi: 10.1176/appi.ajp.2007.07010043 18172018

[26] Shi C Kang L Yao S Ma Y Li T Liang Y. The MATRICS consensus cognitive battery (MCCB): co-norming and standardization in China. Schizophr Res. (2015) 169:109–15. doi: 10.1016/j.schres.2015.09.003, PMID: 26441005 PMC4916953

[27] Bang M Kim KR Song YY Baek S Lee E An SK. Neurocognitive impairments in individuals at ultra-high risk for psychosis: who will really convert? Aust NZ J Psychiatry. (2015) 49:462–70. doi: 10.1177/0004867414561527, PMID: 25425742

[28] Zheng W Zhang QE Cai DB Ng CH Ungvari GS Ning YP. Neurocognitive dysfunction in subjects at clinical high risk for psychosis: a meta-analysis. J Psychiatr Res. (2018) 103:38–45. doi: 10.1016/j.jpsychires.2018.05.001, PMID: 29772485

[29] Chu A Chang WC Chan S Lee E Hui C Chen E. Comparison of cognitive functions between first-episode schizophrenia patients, their unaffected siblings and individuals at clinical high-risk for psychosis. Psychol Med. (2019) 49:1929–36. doi: 10.1017/S0033291718002726, PMID: 30226125

[30] Velthorst E Mollon J Murray RM de Haan L Germeys IM Glahn DC. Cognitive functioning throughout adulthood and illness stages in individuals with psychotic disorders and their unaffected siblings. Mol Psychiatry. (2021) 26:4529–43. doi: 10.1038/s41380-020-00969-z, PMID: 33414498

[31] Garg R Trivedi JK Dalal PK Nischal A Sinha PK Varma S. Assessment of cognition in non-affected full biological siblings of patients with schizophrenia. Indian J Psychiatry. (2013) 55:331–7. doi: 10.4103/0019-5545.120543, PMID: 24459302 PMC3890917

[32] Mucci A Galderisi S Green MF Nuechterlein K Rucci P Gibertoni D. Familial aggregation of MATRICS consensus cognitive battery scores in a large sample of outpatients with schizophrenia and their unaffected relatives. Psychol Med. (2018) 48:1359–66. doi: 10.1017/s0033291717002902, PMID: 29017620

[33] Mondragon-Maya A Ramos-Mastache D Roman PD Yanez-Tellez G. Social cognition in schizophrenia, unaffected relatives and ultra-high risk for psychosis: what do we currently know? Actas Esp Psiquiatr. (2017) 45:218–26. PMID: 29044446

[34] Lee TY Hong SB Shin NY Kwon JS. Social cognitive functioning in prodromal psychosis: a meta-analysis. Schizophr Res. (2015) 164:28–34. doi: 10.1016/j.schres.2015.02.008, PMID: 25749019

[35] Eack SM Mermon DE Montrose DM Miewald J Gur RE Gur RC. Social cognition deficits among individuals at familial high risk for schizophrenia. Schizophr Bull. (2010) 36:1081–8. doi: 10.1093/schbul/sbp026, PMID: 19366983 PMC2963048

